# Computational Analysis of Residue-Specific Binding Free Energies of Androgen Receptor to Ligands

**DOI:** 10.3389/fmolb.2021.646524

**Published:** 2021-03-12

**Authors:** Guangfeng Shao, Jingxiao Bao, Xiaolin Pan, Xiao He, Yifei Qi, John Z. H. Zhang

**Affiliations:** ^1^ Shanghai Engineering Research Center of Molecular Therapeutics and New Drug Development, Shanghai Key Laboratory of Green Chemistry and Chemical Process, School of Chemistry and Molecular Engineering, East China Normal University, Shanghai, China; ^2^ NYU-ECNU Center for Computational Chemistry at NYU, Shanghai, China; ^3^ Department of Chemistry, New York University, New York, NY, United States

**Keywords:** androgen receptor, alanine scanning, interaction entropy, MD simulation, hotspot, GBSA

## Abstract

Androgen receptor (AR) is an important therapeutic target for the treatment of diseases such as prostate cancer, hypogonadism, muscle wasting, etc. In this study, the complex structures of the AR ligand-binding domain (LBD) with fifteen ligands were analyzed by molecular dynamics simulations combined with the alanine-scanning-interaction-entropy method (ASIE). The quantitative free energy contributions of the pocket residues were obtained and hotspot residues are quantitatively identified. Our calculation shows that that these hotspot residues are predominantly hydrophobic and their interactions with binding ligands are mainly van der Waals interactions. The total binding free energies obtained by summing over binding contributions by individual residues are in good correlation with the experimental binding data. The current quantitative analysis of binding mechanism of AR to ligands provides important insight on the design of future inhibitors.

## Introduction

Androgen receptor (AR) is an important target for many diseases including prostate cancer, hypogonadism, muscle wasting, osteoporosis, and benign prostate hyperplasia (Chen et al., 2004; Gao and Dalton, 2007; Dillon et al., 2010; Tan et al., 2015; Li et al., 2019). AR is expressed in many tissues, including prostate, seminal vesicle, testis, epididymis, adrenal gland, skin, skeletal muscle and central nervous system (CNS). Androgen receptor ligands can be divided into androgens (agonists) and antiandrogens (antagonists) depending on whether they activate or inhibit the transcription of AR target genes, or by ligand structure into steroidal and non-steroidal (Gao et al., 2005). Because of the rigid skeleton of steroidal compounds, the majority of recently developed ligands are non-steroidal ligands. Like other nuclear receptors, the androgen receptor is modular in structure and is composed of a N-terminal domain (NTD), a DNA binding domain (DBD), a hinge region, and a C-terminal ligand-binding domain (LBD) (Brinkmann et al., 1989). Most clinically used antiandrogens, such as flutamide (Goldspiel and Kohler, 1990), nilutamide (Kassouf et al., 2003), bicalutamide (Goa and Spencer, 1998), enzalutamide (Nadiminty et al., 2013), apalutamide (Rathkopf et al., 2013), and darolutamide (Fizazi et al., 2018), target LBD.

Understanding of protein-ligand interaction and the quantitative characterization of binding affinity are very important for the discovery, design, and development of drugs (Cheng et al., 2007; Gilson and Zhou, 2007; Jorgensen and Thomas, 2008; Sun et al., 2013; Hu et al., 2020). Although experiments can study the thermodynamic properties of protein-ligand binding, determination of binging affinity is time-consuming, laborious and expensive. Computationally, the molecular mechanics generalized born surface area (MM/GBSA) method is often used to calculate the free energy of protein ligand binding (Massova and Kollman, 1999; Kollman et al., 2000; Moreira et al., 2007; Genheden and Ryde, 2015). Recently we developed a method called interaction entropy (IE) for practical and efficient calculation of entropy in protein-ligand and protein-protein binding (Duan et al., 2016). This method has been used in combination with alanine scanning (Massova and Kollman, 1999) (AS) and MM/GBSA to obtain the residue-specific contribution of each pocket residue (ASE method) (Yan et al., 2017; Liu et al., 2018; Qiu et al., 2018; Zhou et al., 2018; He et al., 2019; Yang et al., 2019; Wang et al., 2020).

In this study, fifteen AR ligands were analyzed with the ASIE method to quantitively characterize the detailed protein-ligand interactions, and the contribution of key binding residues on AR were identified. In addition, there is a strong correlation between the sum of the contributions of residues and the experimental binding free energy.

## Methods

### Molecular Dynamics Simulations

In this study, 15 androgen receptor systems with experimental ki/kd values and complex structures in the Protein Data Bank (Berman et al., 2000) (PDB) were used for MD simulation (Hamann et al., 1998; He et al., 2004; Bohl et al., 2005; Salvati et al., 2005; Sun et al., 2006; van Oeveren et al., 2006; Ostrowski et al., 2007; Bohl et al., 2008; Nirschl et al., 2009; Saeed et al., 2016). The systems were simulated using pmemd.cuda (Case et al., 2005) in AMBER18 (Pearlman et al., 1995; Case et al., 2019) with the ff14SB force field (Maier et al., 2015). For each system, TIP3P (Jorgensen et al., 1983) water model and periodic boundary conditions were used to solvate the complex, and the minimum distance between solute atoms and periodic boundary was set to 12 Å. Furthermore, Sodium and chloride ions were added to neutralize the system. A two-step minimization process was carried out, where only hydrogen atoms were optimized in the first step, and all atoms were optimized in the second step. The system was then slowly heated to 300 K with Langevin dynamics temperature regulation, followed by an equilibration of 500 ps Finally, 10-ns MD simulations were carried out in an NPT ensemble and 25,000 snapshots were saved for further analysis. Five independent replicates were simulated for each system, and the final results were obtained by averaging the calculated values on the five trajectories.

### Binding Free Energy Calculation

We mutated a specific amino acid to alanine, assuming that the mutated alanine contributed little to the binding free energy, and calculated the binding free energy difference before and after the mutation. The free energy difference of a residue *x* mutating to ALA is defined as:

$$\begin{array}{c} \Delta \Delta G_{bind}^{x\to a}=\Delta G_{bind}^{a}-\Delta G_{bind}^{x}\\ =\Delta \Delta G_{gas}^{x\to a}+\Delta \Delta G_{sol}^{x\to a} \end{array}$$ (1)

where the gas-phase:

$$\Delta \Delta G_{gas}^{x\to a}=\Delta G_{gas}^{a}-\Delta G_{gas}^{x}$$ (2)

and solvation components:

$$\Delta \Delta G_{sol}^{x\to a}=\Delta G_{sol}^{a}-\Delta G_{sol}^{x}$$ (3)

In the IE (interaction entropy) approach (Duan et al., 2016), the gas-phase component is computed by

$$\begin{array}{c} \Delta G_{gas}^{x}=\langle E_{\mathrm{int}}^{x}\rangle -T\Delta S_{\mathrm{int}}^{x}\\ =\langle E_{\mathrm{int}}^{x}\rangle +KT\ln\langle e^{\beta \Delta E_{\mathrm{int}}^{x}}\rangle \end{array}$$ (4)

And

$$\Delta G_{gas}^{a}=\langle E_{\mathrm{int}}^{a}\rangle +KT\ln\langle e^{\beta \Delta E_{\mathrm{int}}^{a}}\rangle$$ (5)

Where $E_{\mathrm{int}}^{x}$ and $E_{\mathrm{int}}^{a}$ contain the electrostatic and van der Waals energies between the ligand and residues *x* and Ala. The exponential average was evaluated by discrete time averaging.

$$\langle e^{\beta \Delta E_{\mathrm{int}}^{x}}\rangle =\frac{1}{N}\sum_{i=1}^{N}e^{\beta \Delta E_{\mathrm{int}}^{x}\left(t_{i}\right)}$$ (6)

After that, Eq. 2 becomes:

$$\begin{array}{c} \Delta \Delta G_{gas}^{x\to a}=\Delta \Delta E_{gas}^{x\to a}-T\Delta \Delta S_{gas}^{x\to a}\\ =\langle E_{\mathrm{int}}^{a}\rangle -\langle E_{\mathrm{int}}^{x}\rangle +KT\left[\ln\langle e^{\beta \Delta E_{\mathrm{int}}^{a}}\rangle -\ln\langle e^{\beta \Delta E_{\mathrm{int}}^{x}}\rangle \right] \end{array}$$ (7)

The solvation free energy was calculated by the MM/GBSA method:

$$\Delta G_{sol}=\Delta G_{gb}+\Delta G_{np}$$ (8)

The polarization part $\Delta G_{gb}$ is obtained by the generalized Born (GB) model. The non-polar term $\Delta G_{np}\;$ can be obtained by using the solvent accessible surface area (SASA) formula:

$$\Delta G_{np}=\gamma SASA+\beta$$ (9)

Finally, the free binding energy of protein ligands can be expressed as (Liu et al., 2018; Zhou et al., 2018)

$$\Delta G_{bind}=-\sum_{x}\Delta \Delta G_{bind}^{x\to a}$$ (10)

For each system, 25,000 frames were extracted from the entire 10-ns trajectory at an interval of 400 fs for IE calculation. Hundred frames were uniformly extracted from the 25,000 frames for MM/GBSA calculation with “igb” set to 8 (Ryckaert et al., 1977; Nguyen et al., 2013) because igb = 8 is the latest GB model, and Nguyen et al. proved that the GB-Neck2 model (igb = 8) shows significant improvement in solvation energy and effective radii calculation as compared to GB-OBC (igb = 2, igb = 5) and GB-Neck (igb = 7) (Jorgensen et al., 1983). Following our previous protocol, the dielectric constant for nonpolar, polar, and charged residues are 1, 3, 5, respectively (Hou et al., 2011; Petukh et al., 2015). We also calculated binding energy with the conventional MM/GBSA method for comparison, where the dielectric constant was set to one for all residues. The free energy and its standard deviation were obtained from five free energy values calculated from five independent trajectories.

## Results and Discussion

**FIGURE 1:**
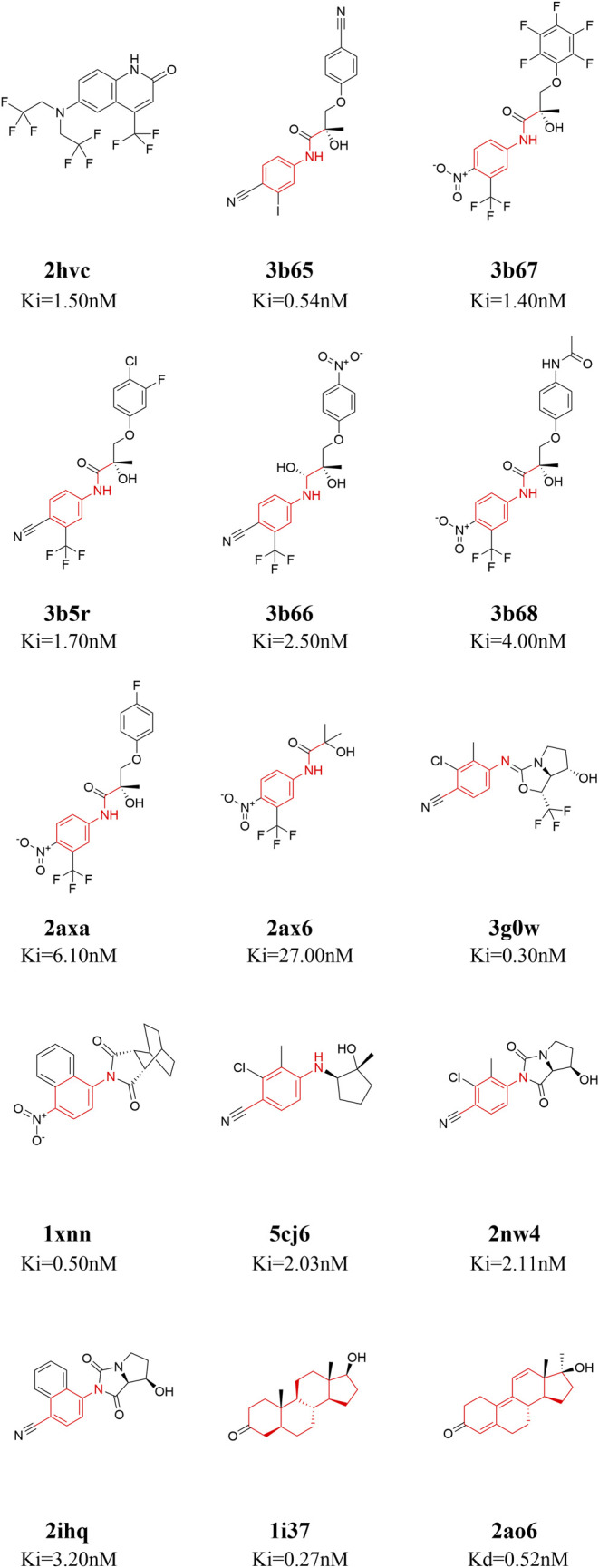
2D structures of the fifteen ligands analyzed in this study. The 15 ligands are divided into four categories according to their structural similarity [cluster 1: 2hvc (Wang et al., 2006); cluster 2: 3b65 (Bohl et al., 2008), 3b67 (Bohl et al., 2008), 3b5r (Bohl et al., 2008), 3b66 (Bohl et al., 2008), 3b68 (Bohl et al., 2008), 2axa (Bohl et al., 2005), 2ax6 (Bohl et al., 2005); cluster 3: 3g0w (Nirschl et al., 2009), 1xnn (Salvati et al., 2005), 5cj6 (Saeed et al., 2016), 2nw4 (Ostrowski et al., 2007), 2ihq (Sun et al., 2006); cluster 4: 1i37 (Sack et al., 2001), 2ao6 (He et al., 2004)], common structures in each cluster are highlighted in red.

Fifteen ligands binding to AR-LBD systems with experimental ki/kd data were used for the binding energy calculations [PDB ID: 1i37 (Sack et al., 2001), 1xnn (Salvati et al., 2005), 2ao6 (He et al., 2004), 2ax6 (Bohl et al., 2005), 2axa (Bohl et al., 2005), 2hvc (Wang et al., 2006), 2ihq (Sun et al., 2006), 2nw4 (Ostrowski et al., 2007), 3b5r (Bohl et al., 2008), 3b65 (Bohl et al., 2008), 3b66 (Bohl et al., 2008), 3b67 (Bohl et al., 2008), 3b68 (Bohl et al., 2008), 3g0w (Nirschl et al., 2009), 5cj6 (Saeed et al., 2016)]. The 2D structures of these 15 small molecules are shown in Figure 1. The 15 molecules are divided into four categories according to their core structures (cluster 1: 2hvc; cluster 2: 3b65, 3b67, 3b5r, 3b66, 3b68, 2axa, 2ax6, these compounds share a N-ethylaniline; cluster 3: 3g0w, 1xnn, 5cj6, 2nw4, 2ihq, these compounds share a p-toluidine; cluster 4: 1i37, 2ao6, both of these are steroidal compounds, Figure 1). There are two steroidal compounds, where the ligand in 1i37 is the natural AR agonist dihydrotestosterone and ligand in 2ao6 is the synthetic steroid agonist. These two compounds have strong binding affinities with AR in the experiment (He et al., 2004; Bohl et al., 2008).

### Hot-Spots Residues in the Complex Structures

**FIGURE 2:**
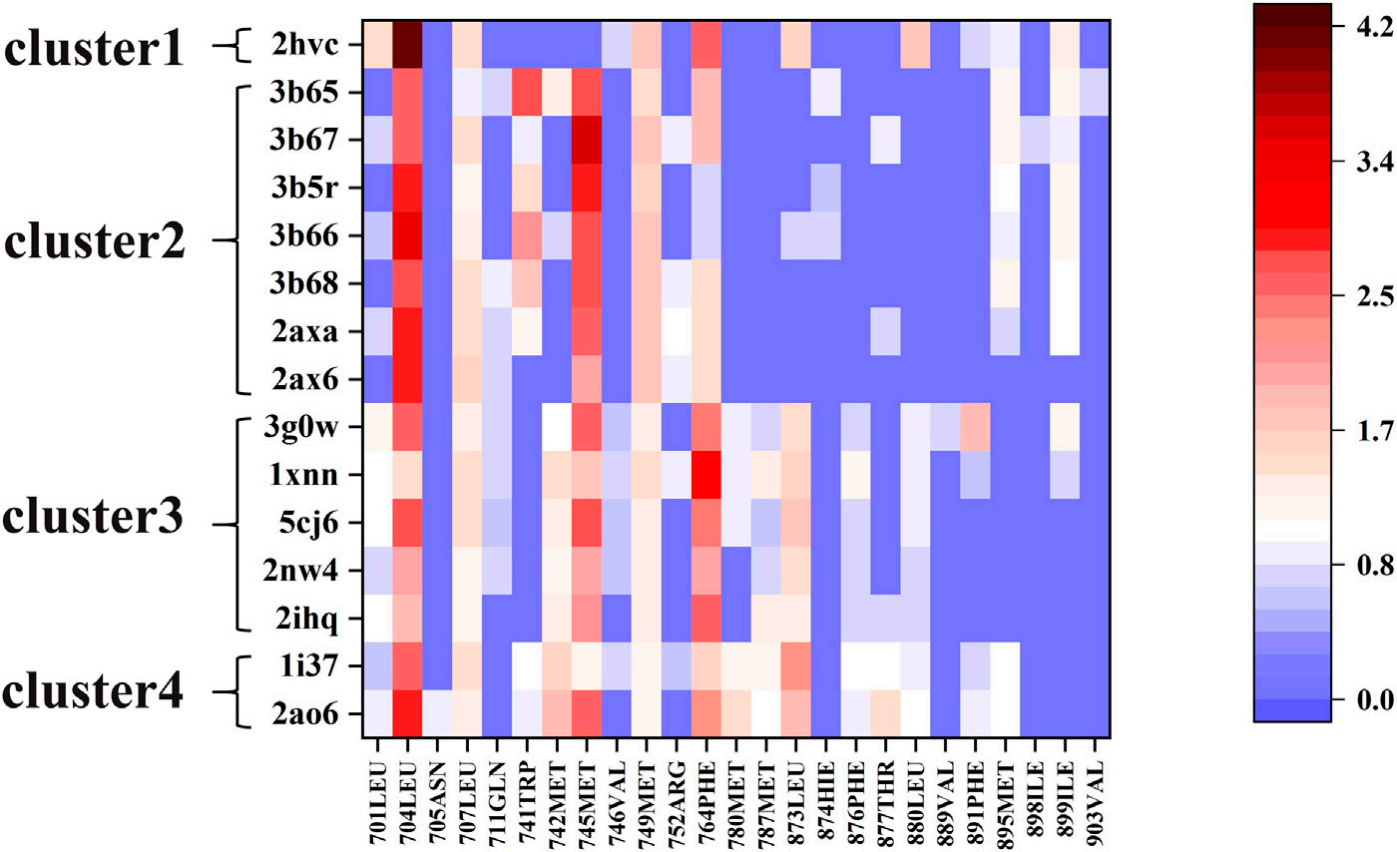
The free energy contributions of each pocket residue in the 15 systems.

We used the ASIE method to quantitatively analyze the contribution of each pocket residue on AR when binds to different ligands. Figure 2 shows the specific binding free energy values of the pocket residues in the 15 systems. First of all, 704LEU, 707LEU, 745MET, 749MET and 764PHE contributed much more to the binding free energy than other residues in these 15 complexes, which are identified as hotspot residues. Secondly, there are clear differences in the contribution of residues to binding energy in these four types of ligands. For example, in cluster 2, residues 741TRP, 895MET, and 899ILE generally have higher binding energy contribution compared to those in cluster 3. In cluster 3, residues 701LEU, 742MET, 780MET, 787MET, 873LEU, 876PHE, and 880LEU have a more prominent binding energy contribution compared to cluster 2.

**FIGURE 3:**
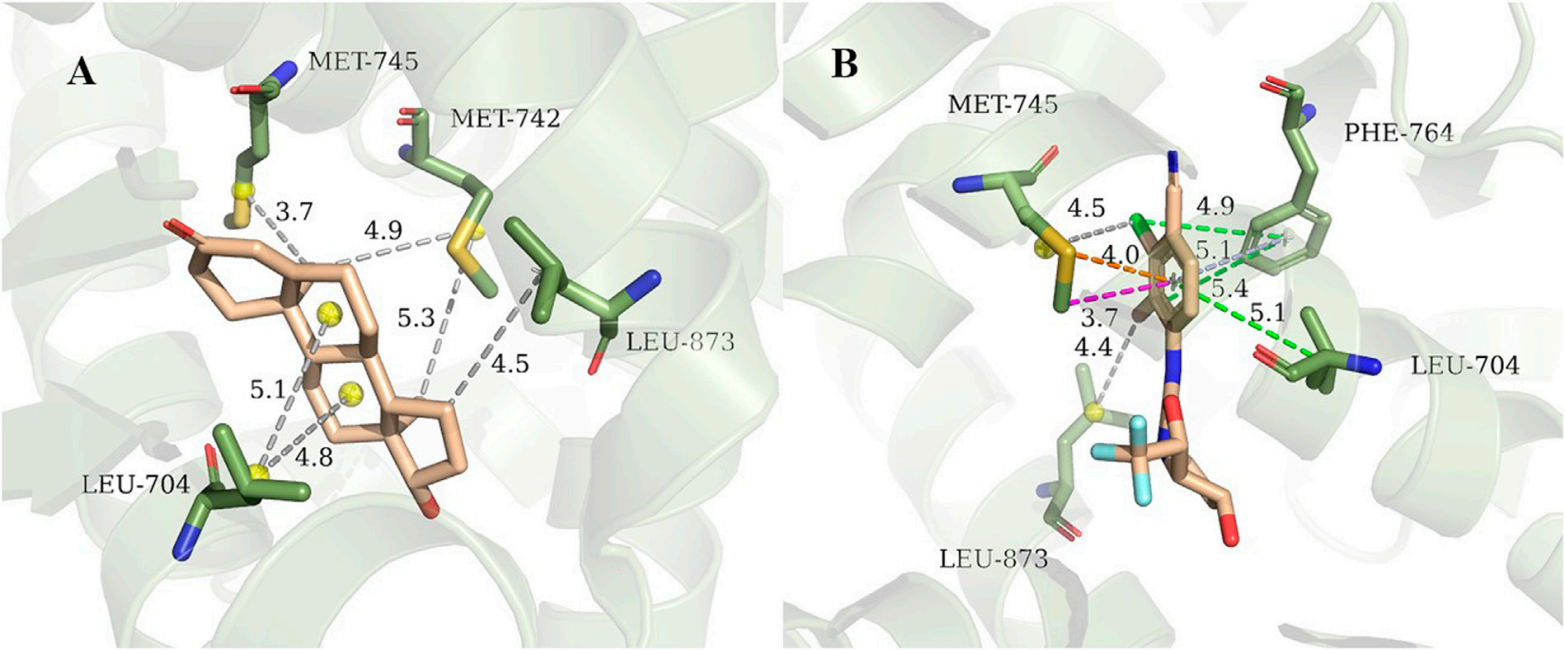
Interaction diagram of 1i37 (Sack et al., 2001) **(A)** and 3g0w (Nirschl et al., 2009) **(B)**. The gray lines represent the alkyl interaction, green lines denote Pi-Alkyl interaction, light blue lines denote Pi-Pi interactions, orange lines denote Pi-Sulfur interactions and magenta lines denote Pi-Sigma interactions.

3g0w **(** Nirschl et al., 2009 **)** and 1i37**(** Sack et al., 2001 **)** from cluster3 and cluster4 are selected for detailed analysis of hot spot residues, because these two systems have the strongest experimental value of binding free energy (Bohl et al., 2008; Nirschl et al., 2009), and the ligands belong to non-steroidal and steroid respectively. In 1i37, 704LEU, 873LEU, 764PHE, 742MET, 745MET, 780MET, 749MET, 787MET, 877THR, 741TRP, and 895MET contribute more than 1 kcal/mol to the total binding energy and are identified as hot residues (Table 1). The crystal structure of 1i37 shows that, LEU704, which is the major contributing residue, forms two Alkyl interactions with the ligand dihydrotestosterone at a distance of 4.8 Å and 5.1 Å (Figure 3A).

**TABLE 1:** Residue-Specific Binding Free Energies (kcal/mol) of 1i37 (Sack et al., 2001) calculated by ASIE.

Residue	ΔΔE_vdw_	ΔΔE_ele_	ΔΔGB	ΔΔNP	ΔH	IE	ΔΔG
704LEU	2.39 ± 0.20	−0.01 ± 0.03	0.39 ± 0.11	0.13 ± 0.02	2.89 ± 0.29	−0.25 ± 0.10	2.64 ± 0.20
873LEU	1.81 ± 0.10	−0.05 ± 0.00	0.53 ± 0.04	0.15 ± 0.01	2.44 ± 0.15	−0.18 ± 0.02	2.25 ± 0.13
764PHE	2.11 ± 0.18	0.64 ± 0.05	−0.08 ± 0.08	0.10 ± 0.01	2.77 ± 0.16	−1.09 ± 0.16	1.68 ± 0.17
742MET	2.00 ± 0.11	0.44 ± 0.02	−0.73 ± 0.08	0.16 ± 0.01	1.87 ± 0.07	−0.25 ± 0.03	1.61 ± 0.06
707LEU	1.53 ± 0.08	0.07 ± 0.02	−0.12 ± 0.02	0.08 ± 0.01	1.57 ± 0.09	−0.15 ± 0.03	1.42 ± 0.08
745MET	1.35 ± 0.08	−0.03 ± 0.06	−0.13 ± 0.08	0.11 ± 0.01	1.29 ± 0.09	−0.09 ± 0.03	1.21 ± 0.09
780MET	1.22 ± 0.11	0.16 ± 0.05	−0.18 ± 0.11	0.12 ± 0.01	1.31 ± 0.06	−0.13 ± 0.01	1.19 ± 0.07
749MET	1.14 ± 0.10	0.26 ± 0.06	0.00 ± 0.08	0.03 ± 0.00	1.43 ± 0.10	−0.25 ± 0.06	1.17 ± 0.09
787MET	1.13 ± 0.06	0.16 ± 0.04	−0.14 ± 0.03	0.08 ± 0.00	1.23 ± 0.06	−0.10 ± 0.01	1.13 ± 0.07
877THR	0.08 ± 0.12	1.77 ± 0.05	−0.51 ± 0.03	0.05 ± 0.00	1.39 ± 0.11	−0.31 ± 0.02	1.07 ± 0.10
741TRP	1.22 ± 0.18	0.12 ± 0.01	−0.14 ± 0.02	0.07 ± 0.01	1.27 ± 0.21	−0.27 ± 0.05	1.00 ± 0.17
895MET	1.01 ± 0.09	0.03 ± 0.04	0.08 ± 0.05	0.08 ± 0.01	1.18 ± 0.08	−0.19 ± 0.02	1.00 ± 0.10
876PHE	1.04 ± 0.05	−0.03 ± 0.03	0.00 ± 0.05	0.08 ± 0.00	1.10 ± 0.03	−0.11 ± 0.01	0.99 ± 0.04
880LEU	0.68 ± 0.04	0.06 ± 0.02	0.15 ± 0.02	0.05 ± 0.00	0.94 ± 0.05	−0.10 ± 0.01	0.84 ± 0.05
746VAL	0.64 ± 0.07	−0.03 ± 0.01	0.26 ± 0.07	0.03 ± 0.01	0.90 ± 0.14	−0.06 ± 0.01	0.84 ± 0.13
891PHE	0.64 ± 0.07	0.50 ± 0.03	−0.24 ± 0.06	0.02 ± 0.00	0.92 ± 0.15	−0.09 ± 0.01	0.83 ± 0.14
701LEU	0.55 ± 0.05	0.05 ± 0.00	0.12 ± 0.02	0.04 ± 0.01	0.76 ± 0.07	−0.07 ± 0.01	0.69 ± 0.08
752ARG	0.56 ± 0.04	1.83 ± 0.07	−1.68 ± 0.05	0.05 ± 0.00	0.76 ± 0.03	−0.11 ± 0.01	0.65 ± 0.03
TOTAL	21.1 ± 0.19	5.93 ± 0.08	−2.43 ± 0.35	1.43 ± 0.02	26.02 ± 0.34	−3.80 ± 0.17	22.22 ± 0.5

For 3g0w, ASIE calculation shows that 745MET, 704LEU, 764PHE, 891PHE, 873LEU, 707LEU, 749MET, 899ILE, 701LEU, and 742MET can be identified as hot residues with a binding free energy contribution of 2.67, 2.60, 2.39, 1.93, 1.54, 1.32, 1.27, 1.20, 1.15, and 1.01 kcal/mol (Table 2). The crystal structure of 3g0w suggests that the interaction between the ligand and the surrounding residues is more abundant than that of 1i37, including the interaction of alkyl, Pi-Alkyl, Pi-Pi, Pi-Sulfur and Pi-Sigma (Figure 3B). The strong contribution of 745MET in calculation is well rationalized in the crystal structure, which shows Pi-Sulfur (4.0 Å), Pi-sigma (3.7 Å) and alkyl (4.5 Å) interactions with the ligand.

**TABLE 2:** Residue-Specific Binding Free Energies (kcal/mol) of 3g0w (Nirschl et al., 2009) calculated by ASIE.

Residue	ΔΔE_vdw_	ΔΔE_ele_	ΔΔGB	ΔΔNP	ΔΔH	IE	ΔΔG
745MET	3.58 ± 0.08	0.45 ± 0.03	−1.25 ± 0.13	0.20 ± 0.02	2.98 ± 0.11	−0.31 ± 0.05	2.67 ± 0.10
704LEU	2.39 ± 0.08	0.19 ± 0.01	0.21 ± 0.06	0.06 ± 0.00	2.85 ± 0.06	−0.24 ± 0.02	2.60 ± 0.07
764PHE	2.46 ± 0.08	0.00 ± 0.01	0.20 ± 0.03	0.08 ± 0.00	2.75 ± 0.08	−0.35 ± 0.03	2.39 ± 0.07
891PHE	1.12 ± 0.03	0.83 ± 0.02	0.13 ± 0.08	0.07 ± 0.01	2.15 ± 0.07	−0.22 ± 0.03	1.93 ± 0.07
873LEU	1.33 ± 0.04	0.03 ± 0.00	0.26 ± 0.02	0.11 ± 0.00	1.73 ± 0.05	−0.20 ± 0.02	1.54 ± 0.05
707LEU	1.32 ± 0.07	0.02 ± 0.01	0.05 ± 0.02	0.07 ± 0.00	1.47 ± 0.08	−0.15 ± 0.01	1.32 ± 0.08
749MET	1.21 ± 0.03	0.45 ± 0.02	−0.16 ± 0.04	0.02 ± 0.00	1.51 ± 0.06	−0.24 ± 0.02	1.27 ± 0.06
899ILE	0.57 ± 0.02	−0.01 ± 0.00	0.60 ± 0.05	0.07 ± 0.00	1.24 ± 0.06	−0.03 ± 0.00	1.20 ± 0.06
701LEU	0.65 ± 0.02	0.10 ± 0.01	0.49 ± 0.06	0.06 ± 0.01	1.30 ± 0.07	−0.15 ± 0.04	1.15 ± 0.03
742MET	2.18 ± 0.11	0.32 ± 0.06	−1.25 ± 0.09	0.16 ± 0.01	1.41 ± 0.17	−0.40 ± 0.07	1.01 ± 0.21
880LEU	0.69 ± 0.04	0.06 ± 0.01	0.22 ± 0.03	0.06 ± 0.00	1.03 ± 0.07	−0.08 ± 0.01	0.94 ± 0.06
780MET	1.37 ± 0.06	1.27 ± 0.11	−1.33 ± 0.09	0.12 ± 0.01	1.43 ± 0.05	−0.52 ± 0.15	0.91 ± 0.15
889VAL	0.13 ± 0.01	0.00 ± 0.00	0.72 ± 0.05	0.00 ± 0.00	0.85 ± 0.06	0.00 ± 0.00	0.84 ± 0.06
787MET	0.93 ± 0.03	−0.04 ± 0.02	−0.07 ± 0.01	0.05 ± 0.00	0.87 ± 0.03	−0.04 ± 0.00	0.83 ± 0.03
711GLN	0.91 ± 0.08	0.30 ± 0.06	−0.33 ± 0.03	0.07 ± 0.00	0.95 ± 0.05	−0.13 ± 0.02	0.82 ± 0.07
876PHE	1.00 ± 0.04	−0.30 ± 0.01	0.11 ± 0.05	0.07 ± 0.00	0.88 ± 0.06	−0.11 ± 0.01	0.77 ± 0.06
746VAL	0.59 ± 0.03	−0.07 ± 0.00	0.17 ± 0.04	0.02 ± 0.00	0.71 ± 0.05	−0.06 ± 0.02	0.65 ± 0.04
TOTAL	22.45 ± 0.15	3.59 ± 0.08	−1.22 ± 0.18	1.28 ± 0.02	26.1 ± 0.32	−3.24 ± 0.35	22.86 ± 0.37

**FIGURE 4:**
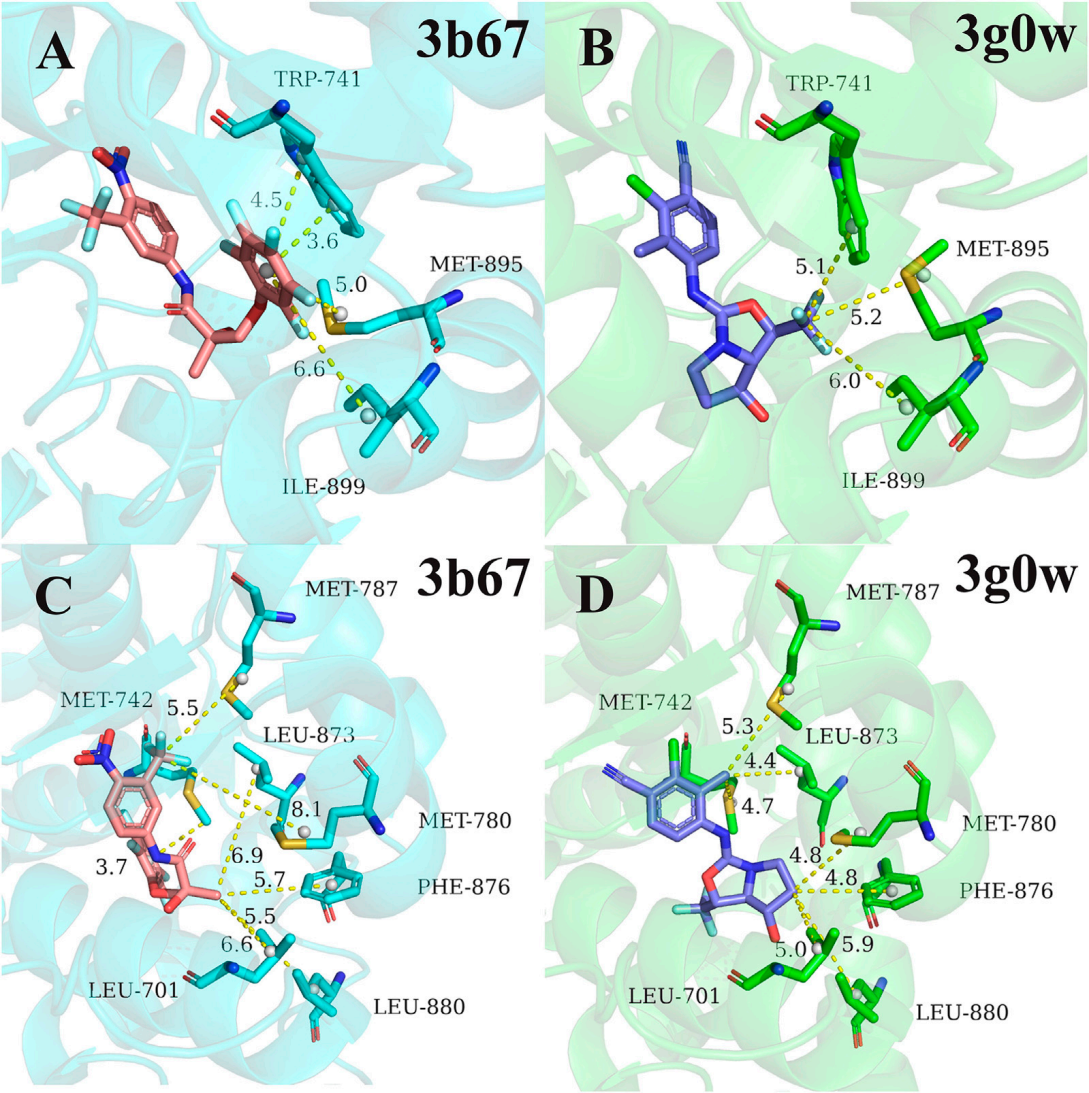
Comparison of pocket residues in 3b67 (Bohl et al., 2008) (cluster2) and 3g0w (Nirschl et al., 2009) (cluster3). The relative position of residues 741TRP, 895MET, 899ILE in 3b67 **(A)** and 3g0w **(B)** with the ligand in the crystal structure. The relative position of the residues 701LEU, 742MET, 780MET, 787MET, 873LEU, 876PHE, 880LEU in the crystal structure to the ligand in 3b67 **(C)** and 3g0w **(D)**. The dashed lines and numbers indicated distance (in Å) between the specific groups of the ligands and residues.

Next, for the different mechanism of those four types of ligands binding to androgen receptor, we analyzed cluster2 and cluster3, among which the most obvious difference was found. As can be seen from Figure 2, residues 741TRP, 895MET and 899ILE in cluster2 has more binding free energy contribution than those in cluster3. Figure 4A,B respectively show the relative positions of these residues and ligands in the crystal structure of 3b67 (Cluster2) and 3g0w (Cluster3). It can be seen that the position of ligands in 3b67 is closer to these three residues than that in 3g0w. In cluster3, 701LEU, 742MET, 780MET, 787MET, 873LEU, 876PHE and 880LEU generally have a stronger binding free energy contribution than those in cluster2. In addition, Figure 4C,D suggest that these residues are generally closer to the ligand in 3g0w (cluster3), which is in good agreement with our calculation results.

The quantitative analysis of these residues specific binding energies provides important clues to design high affinity AR LBD ligands. As shown in Figure 2, the substituents of 2ax6 are shorter than other ligands in Cluster2, AR LBD has fewer residues to interact with it, and its binding free energy is also the lowest in Cluster2. 704LEU has a strong binding free energy contribution in all 15 systems, and in 2hvc, 704LEU has a very high binding energy contribution. This is due to its interaction with the benzene ring on the 2hvc ligand, and the trifluoromethyl on the ligand also interacts with it. Due to its polycyclic structure, cluster4's two steroidal compounds can interact with more residues in AR LBD than the other three types of molecules, which is also of certain reference significance for the design of AR LBD ligand.

### The Total AR-Ligand Binding Energy

We next calculate the total binding energy by summing up the free energy contribution of each residue and compare the results with those obtained using the conventional MM/GBSA method. Table 3 shows the contributions of each energy to the binding free energy of the 15 systems, including enthalpy and entropy. Furthermore, enthalpy is decomposed into van der Waals, electrostatic energy, GB, and nonpolar solvation energy, and it is clear that van der Waals interaction provided most of the enthalpy of each systems. Table 4 shows the computational details of the fifteen systems, including the enthalpy, entropy and binding free energy calculated by ASIE method, the enthalpy calculated by MM/GBSA method and their correlation coefficient, mean absolute error (MAE) and root mean square error (RMSE) with the experimental binding free energy. It can be seen that the binding free energy calculated by ASIE method has a good correlation with the experimental values, and there are also acceptable MAE values and RMSE values.

**TABLE 3:** Free energy components of the binding energies (kcal/mol).

PDB-ID	ΔE_vdw_	ΔE_ele_	ΔGB	ΔNP	ΔH	IE	ΔG	ΔG (exp)^a^
2hvc	12.08 ± 0.19	0.66 ± 0.23	6.88 ± 0.76	0.75 ± 0.01	20.37 ± 0.81	−1.78 ± 0.05	18.59 ± 0.78	12.05
3b65	23.23 ± 0.60	3.75 ± 0.31	−5.16 ± 0.18	1.33 ± 0.02	23.15 ± 0.31	−4.58 ± 0.49	18.57 ± 0.60	12.65
3b67	19.77 ± 0.17	6.38 ± 0.34	−5.91 ± 0.43	1.15 ± 0.04	21.39 ± 0.34	−3.75 ± 0.25	17.64 ± 0.51	12.09
3b5r	17.83 ± 0.52	3.25 ± 0.14	−4.46 ± 0.21	0.92 ± 0.03	17.55 ± 0.34	−3.71 ± 0.25	13.84 ± 0.32	11.97
3b66	23.26 ± 0.27	4.37 ± 0.15	−6.30 ± 0.29	1.48 ± 0.03	22.81 ± 0.39	−5.27 ± 0.34	17.55 ± 0.46	11.75
3b68	19.27 ± 0.32	5.70 ± 0.27	−6.57 ± 0.35	0.99 ± 0.03	19.39 ± 0.40	−3.48 ± 0.33	15.91 ± 0.33	11.47
2axa	20.03 ± 0.28	5.62 ± 0.15	−5.96 ± 0.13	1.17 ± 0.03	20.87 ± 0.24	−4.37 ± 0.27	16.49 ± 0.09	11.22
2ax6	11.83 ± 0.32	3.14 ± 0.15	−2.48 ± 0.28	0.54 ± 0.04	13.03 ± 0.20	−1.44 ± 0.15	11.60 ± 0.24	10.33
3g0w	22.45 ± 0.15	3.59 ± 0.08	−1.22 ± 0.18	1.28 ± 0.02	26.10 ± 0.32	−3.24 ± 0.35	22.86 ± 0.37	13.00
1xnn	24.32 ± 0.28	4.01 ± 1.02	−4.19 ± 0.64	1.28 ± 0.02	25.42 ± 0.69	-3.64 ± 0.18	21.78 ± 0.77	12.70
5cj6	18.78 ± 0.11	1.68 ± 0.12	0.28 ± 0.18	1.10 ± 0.01	21.84 ± 0.21	−2.31 ± 0.10	19.52 ± 0.25	11.87
2nw4	19.55 ± 0.25	1.73 ± 0.14	−3.75 ± 0.14	0.98 ± 0.03	18.50 ± 0.31	−2.83 ± 0.18	15.68 ± 0.42	11.85
2ihq	19.19 ± 0.26	2.71 ± 0.14	−3.85 ± 0.12	0.97 ± 0.01	19.03 ± 0.12	−2.50 ± 0.16	16.52 ± 0.20	11.60
1i37	21.10 ± 0.19	5.93 ± 0.08	−2.43 ± 0.35	1.43 ± 0.02	26.02 ± 0.34	−3.80 ± 0.17	22.22 ± 0.50	13.07
2ao6	21.10 ± 0.27	6.59 ± 0.10	−1.16 ± 0.10	1.22 ± 0.01	27.75 ± 0.33	−2.94 ± 0.09	24.81 ± 0.41	12.68

^a^The experimental value is obtained by using the relation ΔG (exp) = −RT ln Ki/Kd at T = 298K and multiplied by a minus sign.

**TABLE 4:** Binding Free Energies (kcal/mol) calculated by ASIE method compared to the experimental data (kcal/mol).

		ASIE		MM/GBSA^a^	EXP^b^
PDB-ID	ΔH	−TΔS	ΔG	ΔH	ΔG (exp)
2hvc	20.37 ± 0.81	−1.78 ± 0.05	18.59 ± 0.78	8.95 ± 0.83	12.05
3b65	23.15 ± 0.31	−4.58 ± 0.49	18.57 ± 0.60	48.65 ± 0.19	12.65
3b67	21.39 ± 0.34	−3.75 ± 0.25	17.64 ± 0.51	38.58 ± 0.70	12.09
3b5r	17.55 ± 0.34	−3.71 ± 0.25	13.84 ± 0.32	40.28 ± 0.80	11.97
3b66	22.81 ± 0.39	−5.27 ± 0.34	17.55 ± 0.46	40.23 ± 0.57	11.75
3b68	19.39 ± 0.40	−3.48 ± 0.33	15.91 ± 0.33	42.37 ± 1.18	11.47
2axa	20.87 ± 0.24	−4.37 ± 0.27	16.49 ± 0.09	38.14 ± 0.51	11.22
2ax6	13.03 ± 0.20	−1.44 ± 0.15	11.60 ± 0.24	29.56 ± 0.40	10.33
3g0w	26.10 ± 0.32	−3.24 ± 0.35	22.86 ± 0.37	35.02 ± 0.89	13.00
1xnn	25.42 ± 0.69	−3.64 ± 0.18	21.78 ± 0.77	44.45 ± 0.57	12.70
5cj6	21.84 ± 0.21	−2.31 ± 0.10	19.52 ± 0.25	37.77 ± 0.55	11.87
2nw4	18.50 ± 0.31	−2.83 ± 0.18	15.68 ± 0.42	40.59 ± 0.67	11.85
2ihq	19.03 ± 0.12	−2.50 ± 0.16	16.52 ± 0.20	41.39 ± 0.48	11.60
1i37	26.02 ± 0.34	−3.80 ± 0.17	22.22 ± 0.50	47.24 ± 0.52	13.07
2ao6	27.75 ± 0.33	−2.94 ± 0.09	24.81 ± 0.41	48.79 ± 0.27	12.68
R	0.87		0.85	0.34	
MAE	9.53		6.22	27.19	
RMSE	10.03		6.85	28.31	

^a^MM/GBSA results added a minus sign.

^b^The experimental value is obtained by using the relation ΔG (exp) = −RT ln Ki/Kd at T = 298 K and multiplied by a minus sign.

**FIGURE 5:**
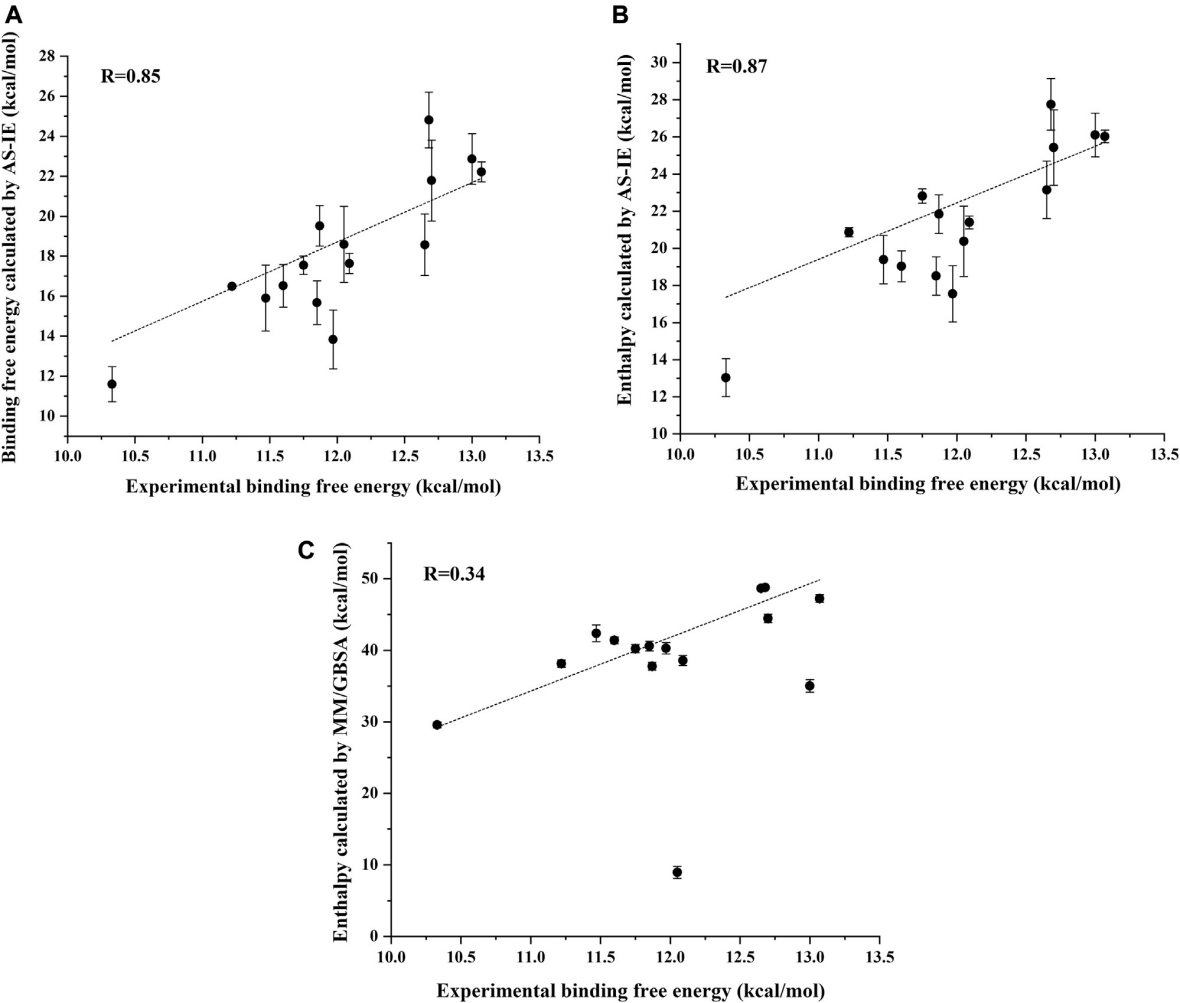
Correlation coefficient R between the experimental binding energy and the binding free energy calculated by ASIE **(A)**, enthalpy calculated by ASIE **(B)** and enthalpy calculated by MM/GBSA method **(C)**.

Figure 5A shows the correlation between the binding free energy obtained from experiments and the binding free energy calculated by ASIE method. The calculated binding energy shows good correlation with the experimental values (R = 0.85), although the experimental values range is very narrow. Figure 5B shows the correlation between the enthalpy calculated by the MM/GBSA method and the experimental binding free energy (R = 0.34). Furthermore, even if the system with the largest error (2HVC) was removed from the MM/GBSA results, the correlation (0.66) between the calculated value and the experimental value was still lower than that of ASIE.

## Conclusion

Androgen receptor (AR) is an important target for many diseases. In our study, we used the ASIE method to quantitatively analyze the contribution of residues when AR binds to different ligands. The residues that contribute most to each ligand are determined. Furthermore, the sum of the contributions of each residue was relatively consistent with the experimental binding energy values. The results of these calculations will be useful for the design and analysis of AR ligands.

## Data Availability

The raw data supporting the conclusions of this article will be made available by the authors, without undue reservation.
